# Effect of a Community Agency–Administered Nurse Home Visitation Program on Program Use and Maternal and Infant Health Outcomes: A Randomized Clinical Trial

**DOI:** 10.1001/jamanetworkopen.2019.14522

**Published:** 2019-11-01

**Authors:** Kenneth A. Dodge, W. Benjamin Goodman, Yu Bai, Karen O’Donnell, Robert A. Murphy

**Affiliations:** 1 Sanford School of Public Policy, Duke University, Durham, North Carolina; 2 Department of Psychiatry and Behavioral Sciences, Duke University School of Medicine, Durham, North Carolina

## Abstract

**Importance:**

Postnatal home visitation to support parenting and infant healthy development is becoming increasingly common based on university efficacy studies, but effectiveness when disseminated by communities is not clear.

**Objective:**

To test implementation and impact of the Family Connects (FC) program when administered by a community agency.

**Design, Setting, and Participants:**

In this randomized clinical trial, births were randomly assigned to receive FC or treatment as usual. Independent evaluation was conducted through parent interviews and review of health and child protective services records. Interviewers were blind to the experimental condition of participants, and participants were blind about the purpose of the interview as an intervention evaluation. A total of 936 consecutive residential births at Duke University Hospital from January 1, 2014, through June 30, 2014, were included. Data were analyzed preliminarily for reporting to funders in early 2015 before all birth-record covariates were scored and were analyzed more comprehensively in mid-2019 after administrative birth and child protective service records became available.

**Interventions:**

The goals of the FC brief universal program were to assess family-specific needs, complete brief interventions, and connect families with community resources. Community agencies and families were aligned through an electronic data system.

**Main Outcomes and Measures:**

Case records documented program penetration and quality. The primary outcome was child protective services investigations for maltreatment. Secondary outcomes were the number of sustained community connections, maternal mental health, parenting behavior, infant well-child care visits and maternal postpartum care compliance, and emergency health care utilization.

**Results:**

Of 936 births, 451 infants (48.2%) were female and 433 (46.3%) were from racial/ethnic minority groups. In all, 456 births (46.5%) were randomized to the intervention and 480 (53.5%) were randomized to the control. All analyses were based on intention to treat. The impact analysis included 158 intervention families and 158 control families. Intervention penetration was 76%, adherence to the protocol was 90%, and independent agreement in scoring (κ) was 0.75. Nurses identified and addressed minor problems for 52% of families and connected an additional 42% to community resources. Analysis of the primary outcome of child abuse investigations revealed a mean (SD) of 0.10 (0.30) investigations for the intervention group vs 0.18 (0.56) investigations for the control group (*b* = −0.09; 90% CI, −0.01 to −0.12; 95% CI, −0.18 to 0.01; *P* = .07). The intervention group’s rate of possible maternal anxiety or depression was 18.2% vs 25.9% for the control group (*b* = −7.70; 90% CI, −15.2 to −0.1; 95% CI, −16.6 to 1.3; *P* = .09).

**Conclusions and Relevance:**

This study indicates that a nurse home visitation program for families of newborns can be implemented by a community agency with high penetration and quality. Other communities could benefit from wider dissemination of the program provided that quality remains strong and evaluation continues.

**Trial Registration:**

ClinicalTrials.gov identifier: NCT01843036

## Key Points

**Question:**

What is the effect of a nurse home visitation program for families with newborns implemented in a community setting on program penetration and fidelity and family outcomes?

**Findings:**

This community-based randomized clinical trial found that the visitation program was implemented with 76% penetration and 90% adherence to the protocol, leading nurses to address minor problems for 52% of families and connect an additional 42% to community resources. Analyses of interviews and administrative records indicated that families assigned to the intervention had more community connections, fewer cases of maternal anxiety or depression, and fewer investigations for child abuse.

**Meaning:**

This study demonstrates successful implementation and decreased child abuse investigations among participants in a postnatal nurse home visitation program implemented at scale in a community setting.

## Teaser

This randomized clinical trial examines the effects of a community agency–administered postnatal nurse home visitation program on child protective services investigations for maltreatment and other maternal and child health-related outcomes.

## Introduction

Home visitation programs promoting healthy development in early childhood have proliferated based on promising empirical evidence.^1^ The federal Maternal, Infant, and Early Childhood Home-Visiting (MIECHV) Program^2^ allocates $400 million annually to programs with positive impact demonstrated through randomized clinical trials (RCTs). Simultaneously, the field is challenged by failures to replicate findings when university-developed programs are implemented by community agencies, known as the *scale-up penalty*. The need for studies of implementation and impact of intervention in community settings is one of the highest priorities in medicine.^3^

The study we report here evaluated implementation and impact of the MIECHV-approved Family Connects (FC) program during its community dissemination following a university RCT.^4^ The FC program is a systems-based approach to supporting families at birth by combining engagement and alignment of community resources with voluntary home visits for all families of newborns, including brief interventions, assessments of family needs, and connections to community resources to meet family-specific needs.^5^ Trained nurses offer mothers one to several home visits for support, clinical assessment, and referrals, as needed. They use motivational interviewing techniques to help families clarify needs in each of 12 empirically based risk domains and to facilitate referrals to community agencies. A community specialist aligns service agencies and makes available to nurses an annotated directory that includes service description, eligibility criteria, fees, and waiting lists. Agencies include formal service providers (eg, substance abuse, maternal depression, parenting support); government and nonprofit programs (eg, housing assistance, childcare subsidies); and paraprofessional parent support groups. An integrated electronic data system documents visits, family needs, and outcomes of referrals to community services. The FC program is unique among MIECHV programs in emphasizing population impact, short-term duration, and low cost (about $500 per family).

A university-based RCT yielded 5 sets of empirical findings.^6,7^ First, the program penetrated a high proportion of the population. Second, the program was implemented with high fidelity. Third, the program achieved its proximal goal of connecting families with community services. Fourth, intervention-assigned families displayed more positive parent mental health and parenting behaviors at age 6 months than control parents. Fifth, hospital records at ages 6 months and 12 months showed that intervention-assigned infants accessed less emergency health care than control infants.

Following the first trial, FC began dissemination to communities across the country.^8^ Concern about possible scale-up penalties on program implementation and impact motivated an RCT of FC when implemented by a community agency. After the first trial, the administration of FC in Durham, North Carolina, transferred to a local nonprofit entity. Funding was provided by county government, Medicaid reimbursement, and philanthropy. Hiring and supervision of nurses and implementation of the program was administered by the local community agency. This study tested 5 assertions undergirding the logic of FC: (1) FC can penetrate a large proportion of the birthing community; (2) FC can be delivered with high quality; (3) families assigned to FC will become more connected to community resources; (4) families assigned to FC will demonstrate better parent mental health and parenting; and (5) families assigned to FC will have lower rates of investigation for child maltreatment and better infant and maternal health care participation and outcomes.

## Methods

### Participants, Intervention Assignment, and Evaluation Design

**Figure 1.**
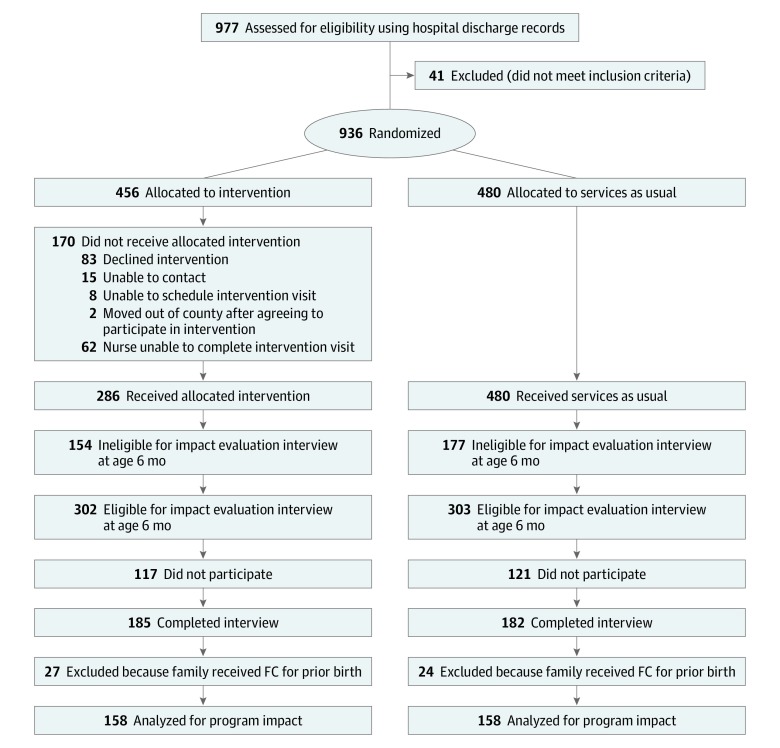
CONSORT Flow Diagram for Family Connects (FC) Randomized Clinical Trial Implementation

All procedures were approved by the Duke Medicine institutional review board. Written informed consent was required of intervention and control participants for outcome evaluation data collection. Intervention families were required to provide consent to participate in the intervention and to allow access to intervention implementation data. Figure 1 provides the CONSORT flow diagram of participants through the trial. From January 1, 2014, through June 30, 2014, of the 977 hospital discharge records that were examined, all 936 county resident births at Duke University Hospital constituted the possible population for assignment to intervention. This sample size was determined based on minimal size needed to detect hypothesized effects. Identical to the design of the first FC RCT,^4^ random assignment to intervention was based on date of birth. Intervention staff members at a community agency attempted to deliver FC to all 456 odd-date births. The 480 even-date births were not approached for FC and instead received treatment as usual (the reverse of the prior trial). Implementation performance was evaluated for the 456 odd-date births. The full trial protocol is available in Supplement 1. Reporting followed the Consolidated Standards of Reporting Trials (CONSORT) reporting guideline.

Implementation followed a detailed manual^4^ that included 3 major components. The first was direct intervention with a family beginning in the birthing hospital and continuing with 1 to 3 postpartum home visits. A nurse assessed and scored family need for intervention for each of 12 key domains assessed during home visits: parent health, infant health, medical home, child care planning, parent-infant relationship, management of infant crying, material supports, family violence, mother’s past experience of maltreatment, maternal depression and anxiety, parental substance abuse, and parental social support from others. Risk for each domain was scored on a 4-point scale: 1 for no risk; 2 for risk that is addressed by a brief intervention; 3 for significant ongoing risk that requires a connection with a community resource to resolve; and 4 for an emergency requiring crisis intervention. For domains scored as 2, the nurse attempted prescribed brief interventions. For domains scored as 3, the nurse facilitated a connection to a community resource for ongoing intervention. The second component of intervention was alignment of community resources to serve families at birth through an electronic directory of agencies. The third component was an integrated data system that documented all services and assessments.

To examine impact of random assignment to intervention, a research assistant blind to experimental condition and unaffiliated with the community agency solicited written informed consent to participate in a research study of infant development, consisting of a single in-home interview at age 6 months (range 4-8 months). These families were blind to the goal of evaluating an intervention. Of the 456 odd-date births, 302 were located and confirmed as eligible (based on Durham residency at age 6 months), and 185 (61% of eligible) completed interviews. Of the 480 even-date births, 303 were located and confirmed as eligible residents, and 182 (60%) completed interviews. For the 15 families with twins, 1 member of each pair was randomly excluded from analysis. Administrative data indicated that 51 families had participated in FC for a prior birth (intervention and control conditions), and these families were excluded from analysis. The final sample included 316 infants (158 intervention and 158 control).

Measures of FC penetration and quality were taken from case records. Baseline covariates for consenting families were collected from hospital discharge records, and measures of intervention impact were taken from parent interviews and administrative records.

### Measures of Baseline Variables

Number of infant birth risks ranged from 0 to 5 and included the following: (1) born at less than 2500 g; (2) less than 37 weeks’ gestational age; (3) birth complications, not specified; (4) substance exposure in utero; and (5) other risk noted, not specified. Cesarean delivery was scored as 1 if yes; otherwise, 0. Multiple births was scored as 1 if yes; otherwise, 0. Adolescent mother was scored as 1 if the mother was aged 19 years or younger; otherwise, 0. Single parent status was scored as 1 if the mother was not married or did not have a residential partner; otherwise, 0. Family Medicaid or no insurance status was scored as 1 if yes; otherwise, 0. Infant sex was scored from the birth record as 1 if female; 0 if male. Infant race/ethnicity was scored from classifications made on the birth record as 1 if minority; otherwise, 0 (additional analyses scored 4 dichotomous variables for race/ethnicity as white, African American, Hispanic, or other).

### Measures of Program Implementation

Records documented the number of families who initiated FC participation and the number who completed the program. Adherence to the manualized protocol was assessed by having an independent expert accompany the nurse on a home visit for 19 randomly selected families and check adherence to each model element. Reliability of scoring of risk between the nurse and the expert was measured by Cohen κ,^9^ which corrects for chance agreement. Clinical records were inspected to score (1) the proportion of families with at least 1 identified need and referral to a community agency (scored as 3 for any of the 12 domains of need); and (2) the proportion of families with identified needs for whom community services were initiated within 1 month after referral. Family consumer satisfaction with the experience of FC was measured during a follow-up telephone call 1 month after case closure. The interviewer asked the parent whether they found the experience helpful (scored 1) or not (scored 0).

### Measures of the Primary Outcome

The primary outcome as listed in the pretreatment registry was child protective services reports for possible child maltreatment (abuse or neglect). State administrative records were examined for all consenting participants for the period from birth through age 24 months to code the number of documented investigations for maltreatment per infant. Reports could also be coded as “substantiation” if proof of maltreatment was documented, but no cases were so coded (primarily owing to a reform that referred cases to clinical services instead of prosecution and therefore did not code documentation as substantiation), so the substantiation variable was not tested.

### Measures of Secondary Outcomes

During the impact interview conducted at infant age 6 months, mothers were asked to name all community resources they had used in the past 3 months, including professional, paraprofessional, and informal resources. The number of community agencies reported was used to score family connections to community resources.

During the interview at infant age 6 months, the mother completed the Edinburgh Postnatal Depression Scale^10^ to detect possible clinical depression (the cut point for depression was a score >10) and the brief Generalized Anxiety Disorder–7 questionnaire^11^ to detect possible clinical anxiety (the cut point for anxiety was a score >5). Maternal mental health problems were scored as 1 if either clinical problem was identified; otherwise, 0.

Parenting decisions and behaviors were assessed by maternal report of positive and negative parenting behaviors^12,13,14^; the modified 21-item Infant Intentionality Questionnaire, which assesses maternal beliefs about intentional infant behaviors^15^; a father-infant relationship quality scale^16^; and a childcare questionnaire.^17^ We scored 5 variables: number of positive parenting behaviors, number of negative parenting behaviors, mother negative attribution of infant intentionality, father-infant relationship quality, and parental decision to use nonparental out-of-home childcare, which was almost always high quality (coded no = 0; yes = 1).

Mothers reported whether the family had complied with a well-baby physician appointment within the past month (coded no = 0; yes = 1). Infant patient records were accessed to score the number of emergency department visits by age 12 months and number of overnight stays in the hospital by age 12 months.

Three variables measured mother’s health care utilization: mother’s report at infant age 6 months assessed mother’s compliance with 6-week postpartum physician visit (coded no = 0; yes = 1). Administrative records were accessed to score the mother’s number of emergency department visits by infant age 12 months and the mother’s number of overnight hospital stays by infant age 12 months.

### Missing Data 

Nineteen participants had at least 1 missing value in a baseline variable. Tests using the Little method indicated that missing cases were not missing completely at random^18^ (χ^2^ = 45.1; *P* = .001). Multiple imputation was used to create 10 data sets in which missing values were imputed from other birth characteristics. Imputed data files were subjected to statistical tests, and estimates were merged.^18,19^

### Statistical Analysis

We next examined equivalence across experimental groups. In Table 1, the columns labeled 1 and 2 list group means for the entire population and the evaluation-eligible groups (ie, those infants still resident in the community and located at age 6 months). Of 11 baseline variables tested, none were significant. Next, we contrasted the evaluation-participating group (column 3) with the entire population (column 1) and found no significant difference across 11 tests. We then tested differences between the intervention (column 4) and control (column 5) evaluation-participating groups and found 2 of 11 variables to be significant. We accounted for pretreatment differences by conducting ordinary least-squares multivariate regression analyses predicting outcomes from random assignment to intervention, with 8 baseline covariates. We also calculated interaction terms between intervention status and the number of birth risk factors. We used linear regression models for continuous outcomes, logistic regression models for binary outcomes, and Poisson models for count variables. Results are reported in Table 2 for all variables, with exact 2-tailed *P* level and 95% and 90% confidence intervals. The text reports variables for which the *P* value is less than .10.

**Table 1.** Comparisons of Preintervention Sample Characteristics for Randomized Clinical Trial Population and Samples

Characteristic	1	2		3		4	5	
	RCT Population (n = 936)	Evaluation Eligible (n = 605)	*P* Value^a^	Evaluation Participating (n = 316)	*P* Value^b^	Intervention Participating (n = 158)	Control Participating (n = 158)	*P* Value^c^
Infant birth characteristics								
Birth risks, mean (SD), No. out of 5	1.0 (1.0)	1.0 (1.0)	.95	1.0 (1.0)	.88	1.1 (1.0)	0.8 (0.9)	<.01
Female, No. (%)	451 (48.2)	293 (48.4)	.93	161 (50.9)	.40	73 (45.9)	88 (56.1)	.07
Mother birth characteristics, No. (%)								
Cesarean delivery	269 (29.6)	173 (29.3)	.90	86 (27.7)	.53	45 (28.7)	41 (26.8)	.71
Multiple births	32 (3.4)	24 (4.0)	.57	15 (4.7)	.28	10 (6.3)	5 (3.2)	.19
Medicaid or no insurance	559 (62.3)	348 (59.9)	.35	197 (64.8)	.44	100 (65.8)	97 (63.8)	.72
Adolescent mother	48 (5.1)	32 (5.3)	.89	21 (6.6)	.31	10 (6.3)	11 (7.0)	.80
Age, mean (SD), y	29.2 (6.0)	29.6 (6.0)	.19	29.4 (6.2)	.63	30.0 (6.2)	28.8 (6.2)	.10
Race/ethnicity, No. (%)								
White	503 (53.7)	327 (54.1)	.91	178 (56.3)	.42	95 (59.7)	83 (52.9)	.22
Black	346 (37.0)	228 (37.7)	.78	120 (38.0)	.75	56 (35.2)	64 (40.8)	.31
Other	87 (9.3)	50 (8.3)	.49	18 (5.7)	.05	8 (5.0)	10 (6.4)	.61
Hispanic	243 (26.0)	146 (24.1)	.42	90 (28.5)	.38	54 (34.0)	36 (22.9)	.03

^a^ The *P* value of column 2 comes from χ^2^ or *t* test between columns 1 and 2.

^b^ The *P* value of column 3 comes from χ^2^ or *t* test between columns 1 and 3.

^c^ The *P* value of column 5 comes from χ^2^ or *t* test between columns 4 and 5.

**Table 2.** Adjusted Group Means and Multivariate Regression Models Testing Main Effect of Treatment and Treatment × Birth Risk Interaction^a^

Outcome	Time to Outcome, mo	Mean (SD), No.		Main Effect				Interaction	
		Intervention (n = 158)	Control (n = 158)	*b*	90% CI	95% CI	*P* Value	*b*	*P* Value
Primary outcome									
Total CPS investigations	24	0.10 (0.30)	0.18 (0.56)	−0.09	−0.01 to −0.12	−0.18 to 0.01	.07	−0.04	.70
Secondary outcomes									
Community support, parent mental health, and parenting									
Total community connections	6	5.72 (2.37)	5.04 (2.69)	0.69	0.27 to 1.11	0.19 to 1.19	.01	−0.02	.70
Possible maternal depression or anxiety, No. (%)	6	29 (18.2)	41 (25.9)	−7.70	−15.20 to −0.12	−16.65 to 1.33	.09	0.01	.98
Positive maternal parenting behaviors	6	4.40 (0.57)	4.45 (0.52)	−0.05	−0.14 to 0.05	−0.16 to 0.07	.42	−0.06	.34
Negative maternal parenting behaviors	6	0.07 (0.18)	0.09 (0.27)	−0.02	−0.06 to 0.02	−0.07 to 0.03	.47	0.03	.33
Negative intentionality beliefs	6	0.13 (0.31)	0.16 (0.31)	−.03	−0.09 to 0.02	−0.10 to 0.03	.34	0.00	.96
Father-infant relationship quality	6	2.13 (0.68)	2.19 (0.67)	−0.07	−0.19 to 0.06	−0.22 to 0.08	.37	0.00	.97
Family uses non-parental childcare, No. (%)	6	86 (54.3)	70 (44.0)	10.30	1.42 to 19.23	−0.31 to 20.92	.06	0.05	.85
Infant health care utilization									
Pediatric appointment within past mo, No. (%)	6	89 (56.4)	97 (61.1)	4.70	−13.82 to 4.49	−15.57 to 6.24	.40	0.29	.22
Emergency department visits	12	0.51 (0.84)	0.64 (1.10)	−0.13	−0.30 to 0.05	−0.34 to 0.09	.25	−0.26	.02
Overnights in hospital	12	0.96 (4.04)	0.65 (4.31)	0.31	−0.43 to 1.05	−0.57 to 1.19	.49	0.95	.05
Maternal health care utilization									
Completed 6 week postpartum visit, No. (%)	6	143 (90.3)	132 (83.8)	6.44	0.56 to 12.34	−0.62 to 13.51	.07	0.64	.09
Emergency department visits	12	0.40 (1.14)	0.20 (0.64)	0.21	0.04 to 0.37	0.01 to 0.40	.04	−0.21	.44
Overnights in hospital	12	0.13 (0.61)	0.12 (0.67)	−0.01	−0.11 to 0.13	−0.13 to 0.15	.91	0.20	.09

Abbreviation: CPS, child protective services.

^a^ All analyses conducted using 10 multiply imputed data sets to address missing data. All models included the baseline characteristics listed in Table 1 as covariates.

## Results

### Program Implementation

#### Program Penetration

Among intervention-assigned families, 76% were successfully approached, gave informed consent, and began participation in FC, compared with 80% in the university trial. Among those who began participation, 82% completed the program, compared with 86% in the first trial.

#### Program Quality and Family Satisfaction

Adherence to the FC protocol was 90%, which was higher than the 84% scored in the university trial. Independent rater agreement in scoring the 12 risk factors was κ = 0.75, which was higher than κ = 0.69 achieved in the first trial. Nurses identified and addressed minor family needs for 52% of all possible cases, compared with 49% in the university trial. All 192 family members responded that they found the FC experience helpful.

#### Family Connections to Community Resources

Nurses identified major family needs (scored as 3) and tried to initiate community referral and service for 42% of all families, compared with 45% in the first trial. According to the 1-month follow-up inquiries, nurses were successful in getting high-need families to initiate services in 83% of all cases, compared with 79% in the university trial.

### Program Impact 

#### Primary Outcome 

Table 2 lists group means and test results for ordinary least-squares analyses of intervention main effects and interactions for all variables. The intervention group had a mean (SD) rate of child protective services investigations of 0.10 (0.30), and the control group had a mean (SD) rate of 0.18 (0.56) (*b* = −0.09; 90% CI, −0.01 to −0.12; 95% CI, −0.18 to 0.01; *P* = .07).

#### Impact on Secondary Outcomes

Families assigned to the intervention group had a mean (SD) of 5.72 (2.37) community connections, and the control group had a mean (SD) of 5.04 (2.69) community connections (*b* = 0.69; 90% CI, 0.27-1.11; 95% CI, 0.19-1.19; *P* = .01). The rate of maternal mental health problems was 18.2% for the intervention group and 25.9% for the control group (*b* = −7.70; 90% CI, −15.2 to −0.1; 95% CI, −16.6 to 1.3; *P* = .09). The use of nonparental childcare was 54.3% for the intervention group and 44.0% for the control group (*b* = 10.30; 90% CI, 1.42 to 19.23; 95% CI, −0.31 to 20.92; *P* = .06).

**Figure 2.**
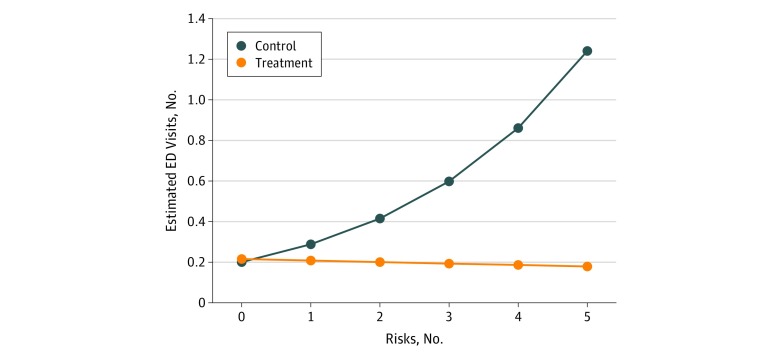
Interaction Between Intervention Status and the Number of Birth Risks for the Cumulative Number of Emergency Department Visits by Age 12 Months Data represent regression-fitted point estimates for treatment and control groups for each level of pretreatment health risk defined by birth records. Risks included the following: (1) born at less than 2500 g; (2) less than 37 weeks’ gestational age; (3) birth complications, not specified; (4) substance exposure in utero; and (5) other risk noted, not specified.

The interaction effect between intervention status and the number of birth risks indicated that assignment to intervention was associated with fewer emergency department visits but only as the number of birth risks increased (*b* = −0.26; *P* = .02). This interaction effect is depicted in Figure 2. For hospital overnights, the interaction effect indicated a higher rate for FC-assigned infants than control infants, but only as the number of birth risks increased.

Mothers in the intervention group had a 6-week postpartum health care compliance rate of 90.3% vs the control group rate of 83.8% (*b* = 6.44; 90% CI, 0.6 to 12.3; 95% CI, −0.62 to 13.51; *P* = .07). Mothers in the intervention group had a mean (SD) number of emergency department visits of 0.40 (1.14) vs. 0.20 (0.64) for the control group (*b* = 0.21; 90% CI, 0.04-0.37; 95% CI, 0.01-0.40; *P* = .04). Mothers in the intervention group had more hospital overnights but only as the number of birth risks increased (*b* = 0.20; *P* = .09).

## Discussion

This randomized clinical trial found that when implemented by a community agency, the FC program achieved a high level of penetration to the full population of birthing families with a high degree of fidelity to the program model. It achieved its proximal goals of identifying family needs, responding to those needs with high parental satisfaction, and connecting families to community resources for major needs. Families randomly assigned to FC had 44% lower rates of investigation for child maltreatment by child protective services. The FC-assigned families had more connections to community resources and better mental health. Not all analyses confirmed program goals or met statistical significance criteria, indicating the need for caution and continued evaluation of this program as it is disseminated to new communities.

As a public health program aiming for population impact, the first goal of FC is to reach as many families giving birth as possible. The community agency was able to reach about as many families as had been reached in the university trial with similar follow-through to program completion, despite forces cited by Welsh et al^20^ that would otherwise promote degradation. The second goal of FC is to deliver the program with high adherence and fidelity to the manualized protocol. The community agency was able to reach similar quality as exhibited in the university trial.

The third goal of FC is to identify and respond to clinically assessed family needs with connections to community resources. Nurses were able to identify needs and respond through brief interventions or connections to community resources, and have those connections continue for at least 6 months at rates that were similar to those in the prior trial. This function might be called primary psychosocial care, similar to the primary health care function served by pediatricians. Well-baby pediatric health care is universally delivered, assessments are completed, modest interventions respond to clinically identified needs, and referrals are made to specialists when warranted. The FC intervention is a step toward a new system of universal primary psychosocial care for families of newborns.

The primary hypothesis guiding this study is that if the FC program can reach most families with high quality and connect them to community supports, then impact will be observed on rates of child maltreatment. We found positive impact on lowering official rates of child maltreatment investigations, by 44%.

It was secondarily hypothesized and found that the FC program had a positive impact on maternal mental health, replicating the previous trial. In contrast with the previous trial, there was no impact on parenting behavior or nonparental care. Further scrutiny is necessary to understand whether subtle degradation in the aspect of the program related to parenting behavior occurred and could be corrected or the generalizable impact on parenting behavior is fragile. The impact of the FC program on infant and maternal health care utilization was mixed. As birth risk increased, assignment to FC was associated with fewer emergency department visits and more hospital overnights. This pattern was not hypothesized, nor was it found in any other trial, and it may not be reliable. Finally, assignment to FC was associated with higher maternal emergency department visit rates, possibly reflecting greater attentiveness by mothers to their own health needs as a function of FC.

### Limitations

This study has some limitations. The findings of this study highlight both replicated and nonreplicated effects of the FC program when implemented by a community agency. One limitation of the study is that it occurred in only 1 community; thus, it will be important to conduct additional studies with new communities, contexts, and populations. Another limitation is that the active ingredients and mediational processes through which the intervention has impact were not tested and are left to further inquiry. A third limitation is that the evaluation was conducted by the same team of investigators that evaluated the first trial; independent evaluators should be identified for future trials.

## Conclusions

This trial indicates that it is possible to transfer delivery of a university-based universal postnatal nurse home visitation program to community leadership while maintaining high program penetration, quality, and impact. The FC program substantially reduced child maltreatment investigations through the first 24 months of life. We recommend continued dissemination of this public health program in new communities when accompanied by ongoing implementation evaluation and, when possible, impact evaluation.

## Notes

Supplement 1. **Trial Protocol**

Supplement 2. **Data Sharing Statement**

## References

[1] Haskins R, Margolis G Show Me the Evidence: Obama’s Fight for Rigor and Evidence in Social Policy. Washington, DC: Brookings Institution; 2015.

[2] Health Resources & Services Administration. Maternal, Infant, and Early Childhood Home-Visiting (MIECHV) Program. https://mchb.hrsa.gov/maternal-child-health-initiatives/home-visiting-overview. Accessed February 1, 2019.

[3] Simons DJ The value of direct replication. Perspect Psychol Sci. 2014;9(1):-. doi:10.1177/1745691613514755 26173243

[4] Dodge KA, Goodman WB, Murphy RA, O’Donnell K, Sato J Randomized controlled trial of universal postnatal nurse home visiting: impact on emergency care. Pediatrics. 2013;132(suppl 2):S140-S146. doi:10.1542/peds.2013-1021M 24187116 PMC3943376

[5] Dodge KA, Goodman WB, Murphy R, O’Donnell K, Sato J Toward population impact from home visiting. Zero Three. 2013 b;33(3):17-23. 23526864 PMC3606025

[6] Dodge KA, Goodman WB, Murphy RA, O’Donnell K, Sato J, Guptill S Implementation and randomized controlled trial evaluation of universal postnatal nurse home visiting. Am J Public Health. 2014;104(1)(suppl 1):S136-S143. doi:10.2105/AJPH.2013.301361 24354833 PMC4011097

[7] Goodman WB, Dodge KA, Bai Y, O’Donnell KJ, Murphy RA Randomized controlled trial of Family Connects: Effects on child emergency medical care from birth to 24 months [published online September 3, 2019]. Dev Psychopathol. doi:10.1017/S0954579419000889 31477190 PMC7061922

[8] Goodman WB, Christopoulos C, Quinn J Evaluation of the Family Connects Northeast Program in the North Carolina Race to the Top Early Learning Transformation Zone: Final Report. Durham, NC: Duke University; 2016.

[9] Cohen J Statistical Power for the Behavioral Sciences. 2nd ed Hillsdale, NJ: Lawrence Erlbaum & Associates; 1988.

[10] Cox JL, Holden JM, Sagovsky R Detection of postnatal depression: development of the 10-item Edinburgh Postnatal Depression Scale. Br J Psychiatry. 1987;150:782-786. doi:10.1192/bjp.150.6.782 3651732

[11] Spitzer RL, Kroenke K, Williams JB, Löwe B A brief measure for assessing generalized anxiety disorder: the GAD-7. Arch Intern Med. 2006;166(10):1092-1097. doi:10.1001/archinte.166.10.1092 16717171

[12] Durham Family Initiative DFI Cross-Site Interview. Durham, NC: Center for Child & Family Policy, Duke University; 2008.

[13] Lounds JJ, Borkowski JG, Whitman TL; Centers for the Prevention of Child Neglect Reliability and validity of the mother-child neglect scale. Child Maltreat. 2004;9(4):371-381. doi:10.1177/1077559504269536 15538036

[14] Straus MA, Hamby SL, Finkelhor D, Runyan D The Parent-Child Conflict Tactics Scales (PCCTS), Form A. Durban: Family Research Laboratory, University of New Hampshire; 1995.

[15] Berlin LJ, Dodge KA, Reznick JS Examining pregnant women’s hostile attributions about infants as a predictor of offspring maltreatment. JAMA Pediatr. 2013;167(6):549-553. doi:10.1001/jamapediatrics.2013.1212 23588683 PMC3753676

[16] Center for Research on Child Wellbeing The Fragile Families and Child Wellbeing Study (survey of new parents): mothers’ baseline survey. http://www.fragilefamilies.princeton.edu/documentation.asp. Accessed May 23, 2008.

[17] Bates JE, Marvinney D, Kelly T, Dodge KA, Bennett DS, Pettit GS Childcare history and kindergarten adjustment. Dev Psychol. 1994;30(5):690-700. doi:10.1037/0012-1649.30.5.690

[18] Little RJ, Rubin DB Statistical Analysis With Missing Data. New York, NY: John Wiley & Sons; 2014.

[19] Garson GD Missing Values Analysis and Data Imputation. Asheboro, NC: Statistical Associates Publishers; 2015.

[20] Welsh BC, Sullivan CJ, Olds DL When early crime prevention goes to scale: a new look at the evidence. Prev Sci. 2010;11(2):115-125. doi:10.1007/s11121-009-0159-4 19936922

