# Supplementary material for: Effect of a Community Agency–Administered Nurse Home Visitation Program on Program Use and Maternal and Infant Health Outcomes: A Randomized Clinical Trial
Source: JAMA Netw Open. 2019 Nov 1;2(11):e1914522. doi: 10.1001/jamanetworkopen.2019.14522 (PMC6826644; doi:10.1001/jamanetworkopen.2019.14522)
Supplement: Supplement 1. — Trial Protocol [file jamanetwopen-2-e1914522-s001.pdf]

DUHS IRB Application (Version 1.2)

General Information

***Please enter the full title of your protocol:**

Impact Evaluation of a Durham Connects RCT Replication

***Please enter the Short Title you would like to use to reference the study:**

Project KIDS
* This field allows you to enter an abbreviated version of the Study Title to quickly identify this study.

Add Study Organization(s):

List Study Organizations associated with this protocol:

Primary Dept?	Department Name
◆	**DUHS** - Duke Default Department

Assign key study personnel (KSP) access to the protocol

*** Please add a Principal Investigator for the study:**

(Note: Before this study application can be submitted, the PI MUST have completed CITI training)

Dodge, Kenneth

3.1 If applicable, please select the Key Study personnel: (Note: Before this study application can be submitted, all Key Personnel MUST have completed CITI training)

A) Additional Investigators, Primary Study Coordinator (CRC), and the Primary Regulatory Coordinator (PRC):

Goodman, William
Primary Regulatory Coordinator

B) All Other Key Personnel

Bai, Yu
Statistician
Edwards, Matthew
Data Manager
Martin, Melissa
Study Coordinator
Nousak, Philip
Data Manager

Quinn, Jeffrey
Collaborator
Reeves, Krysta
Graduate Student
Skandar, Alexa
Study Coordinator

***Please add a Study Contact:**

Dodge, Kenneth
Goodman, William
Martin, Melissa
Skandar, Alexa

The Study Contact(s) will receive all important system notifications along with the Principal Investigator. (e.g., The study contact(s) are typically the Principal Investigator, Study Coordinator, and Regulatory Coordinator.)

Oncore

Please select the Library for your Protocol:

This field is used in OnCore. Determines the Reference Lists, Forms, Protocol Annotations, Notifications, and Signoffs available for the protocol. Protocols that require reporting to the NCI (National Cancer Institute), must select the Oncology library.

- ○ Oncology
- ◉ Non-Oncology

Protocol Application Type

Select the type of protocol you are creating:

Please see additional criteria and information in the policy titled “Reliance on the IRB of Another Institution, Organization, or an Independent IRB" on the **IRB web site**.

- ◉ Regular Study Application - Most common. The IRB will determine if the study is eligible for expedited review or requires full board review upon submission.
- ○ Application for Exemption from IRB Review - Includes Exempt, Not Human Subject Research, & Not Research.
- ○ External IRB Application - Any study using an external IRB as the IRB-of-Record.
- ○ Trainee Research While Away from Duke - Research conducted by medical students overseen by the Office of Curriculum & other student/trainee research away from Duke.
- ○ Individual Patient Expanded Access, Including Emergency Use - Use of an investigational product under expanded access, including emergency use of an investigational drug or biologic or emergency use of an unapproved device.

Conflict of Interest

Do any of the participating study investigators or other key personnel (or their immediate family/significant other) have a financial or intellectual interest in, or are receiving compensation from, the sponsor or the drugs, devices or technologies used in this research?

○ Yes ◉ No

Are any key personnel an inventor of any of the drugs, devices or technologies used in this research?

Yes No

Do any key personnel have or anticipate (within the year) any financial relationships (e.g., consulting, speaking, advisory boards, patents, equity, options) that could be perceived to overlap or present a conflict of interest with the current research?

Yes No

Do any key personnel have a conflict of interest management plan (issued by the Duke University School of Medicine Research Integrity Office) with this company?

Yes No

Oversight Organization Selection

CRU (Clinical Research Unit) or Oversight Organization Selection:

Please select the CRU.

Campus Oversight Organization/DOCR

The Clinical Research Unit that takes responsibility for this study.

- More information on CRUs can be found on the Duke Office of Clinical Research (DOCR) website, **http://docr.som.duke.edu**
- Questions concerning CRU selection should be directed to docr.help@dm.duke.edu.
- For questions about the Campus Oversight Organization, please visit **Campus Oversight Organization**.

List all Key Personnel on the study who are outside Duke:

- **Note:** You will also need to attach the documentation of Human Subjects Certification for each individual, if they have completed the certification somewhere other than Duke.
- **If outside key personnel will have access to Duke PHI, a data transfer agreement AND external site IRB approval (or IRB authorization agreement) will be needed.** See HRPP policy **Use of Research Data by Former Duke Students or Former Duke Faculty and Employees**
- In the panel below, “PHI” is Protected Health Information.

Entry 1

**Name**	
**Study Role**	
**Email Address**	
**Institution / Organization**	
**Will he/she have access to Duke P.H.I.?**	Yes No
**Is he/she an unpaid volunteer at Duke on the study?**	Yes No

Indicate the Protocol source below:

The protocol source is the author of the protocol. If the protocol is a joint authorship between multiple sources, select the primary author.

An IRB fee may be assessed for all research that is supported by for-profit entities and requires full board review. For additional information, see the **IRB fees section of the IRB web site**

- (selected) PI initiated
- ( ) Commercial / Industry (for-profit entity) initiated
- ( ) Federal Government initiated
- ( ) Cooperative Group Initiated
- ( ) Foundation (non-profit group) initiated
- ( ) Other

Sponsor and Funding Source

Add all funding sources for this study:

View Details	Sponsor Name	Sponsor Type	Contract Type:	Project Number	Award Number
⊟	National Institute of Child Health and Human Development	Federal Government	Grant		

Sponsor Name:	National Institute of Child Health and Human Development
Sponsor Type:	Federal Government
Sponsor Role:	Funding
**Grant/Contract Number:**	5R01 HD069981
**Project Period:**	From:04/01/2012 to:04/30/2023
**Is Institution the Primary Grant Holder:**	Yes
Contract Type:	Grant
Project Number:	
Award Number:	
Grant Title:	
PI Name:<br>(If PI is not the same as identified on the study.)	
Significant Discrepancy:	

Is this a federally funded study?

(selected) Yes ( ) No

As part of this study, will any samples or PHI be transferred to/from Duke to/from anyone other than the Sponsor, a Sponsor subcontractor, or a Funding Source?

( ) Yes (selected) No

Is the Department of Defense (DOD) a funding source?

( ) Yes (selected) No

For Federally funded studies:

Is your funding subject to, and does it comply with, the funding agency's policy for data sharing?

○ Yes ◉ No

Enter the Grant Number or Other Federal Agency Proposal or Application Number:

5R01 HD069981

Note: The Federal Funding Agency ID Number is the Sponsor's grant number assigned to your project and available on your Notice of Award (example: R01HL012345).

If known, enter the SPS (Sponsored Projects System) number if applicable:

2031814

In the Initial Submission Packet, attach the following:
(1) The entire grant, or an explanation of why a grant is not needed.
(2) NIH institutional Certificate form related to data sharing (if applicable).

The entire grant is needed so that it may be reviewed against the protocol for concordance.

Have you successfully synced your protocol to OnCore by clicking the 'Sync Data Over API' button at the top of this page?

Please verify that the protocol has been created in OnCore before submitting this application for PI Signoff.

◉ Yes, I synced my protocol to OnCore and verified it was successfully sent by logging into OnCore.
○ I may have forgotten! I'll click it again right now, just to be sure, and verify it was successfully sent by logging into OnCore.

Mobile Devices and Software

Does this study involve the use of a software or a mobile application?

○ Yes ◉ No

List all software, including third party (non-Duke) and mobile apps, that will be utilized for ascertainment, recruitment, or conduct of the research/project: (eg, MaestroCare, DEDUCE):

Multi-site Research

Is this a multi-site study?

○ Yes ◉ No

Complete for each site if Duke is the Primary grant awardee or coordinating center:

Entry 1

**Site Name:**	
**City:**	
**State/Province:**	

**Country:**	
	**Site Contact Information**
**Primary Contact Name:**	
**Primary Contact Phone:**	
**Primary Contact Email:**	
	**Site Details**
**Does the site have an IRB?**	○ Yes ○ No
**Site IRB approval expiration date:**	
**If date not provided, explanation of why:**	
**Has the site granted permission for the research to be conducted?**	○ Yes ○ No
**Does the site plan to rely on the DUHS IRB for review?**	○ Yes ○ No
**What is the status of the study at this site?**	○ Open<br>○ Closed
**Site approval letters or site personnel lists:**	Attach site approval letters, site closure letterS (if applicable), or site personnel lists in the Initial Submission Packet.

Research Abstract

Please type your Research Abstract here:

The Research Abstract should summarize the main points of your study in one paragraph. The following guidelines may help you:

1. Purpose and objective (1-2 sentences)
2. Study activities and population group (2-4 sentences)
3. Data analysis and risk/safety issues (1-2 sentences)

The current study proposes an independent impact evaluation that will assess the impact of the Durham Connects community-wide newborn nurse home visiting program on: (1) child maltreatment; (2) mother and infant health and health care utilization, (3) parenting, parent-child relationship quality, and child care utilization and development and parenting; (4) family service receipt and connection to community resources; and (5) early childhood education. Participants (n=1800) will include families of all resident Durham County births from January 1, 2014 – June 30, 2014 at Duke and Durham Regional hospitals. A research interview will occur when infants are between 4-8 months of age, including a maternal interview about about parenting and parent-child relationship quality, mother and child health and health care utilization, family service receipt, and maternal mental health, as well as staff observations of the home environment. Mothers will also be asked for permission to access mother and infant administrative records, including hospital/emergency department, DSS, birth, Department of Public Instruction, and Durham Connects program records, which will be reviewed for child health and family service needs and receipt, as well as mother and child health and well-being until child age 12 years. We anticipate that about half of the participants will have participated in the Durham Connects Program and about a quarter will have participated in other family community services. Analyses will examine child and family outcomes and service receipt as a

function of previous service participation (e.g., Durham Connects, other services). In comparison to the anticipated benefits to participants and others, the risks in this study are considered minimal, including only some discomfort and/or self-consciousness for participants.

Research Summary

State your primary study objectives

The purpose of the proposed study is to evaluate the effects of the replication of a community-wide nurse home visiting program called "Durham Connects" on maternal and infant health and well-being. This program recruits mothers in the hospital at birth, provides up to three home visits, establishes connections between the family and primary health care or other community providers, and then follows up one month later to confirm these referrals. For the time period between January 1, 2014 and June 30, 2014, Durham Connects is being implemented to half the community during this time (approximately 1800 total births), with all odd-day births being offered the program, and all even-day births not being offered the program (Durham Connects Evaluation, Pro00017478). The current study proposes an independent impact evaluation that will assess the impact of Durham Connects on: (1) child maltreatment; (2) mother and infant health and health care utilization, (3) parenting, parent-child relationship quality, and child care utilization and development and parenting; (4) family service receipt and connection to community resources; and (5) early childhood education. The aforementioned outcomes of the proposed project are all important and innovative contributions that are necessary to advance the field of early childhood programs. By determining whether previously identified positive program impacts (described below) can be replicated in an independent randomized controlled trial (RCT), the project seeks to reveal opportunities for transformational change to fundamentally improve population-level health and well-being outcomes for infants and families.

State your secondary study objectives

Please select your research summary form:

Standard Research Summary Template

This is the regular (generic) research summary template which is required for all regular applications (unless your protocol fits under the other research summary templates in this category). Use of these instructions is helpful for ensuring that the research summary contains all necessary elements.

Standard Research Summary

Purpose of the Study

- Objectives & hypotheses to be tested

The current study proposes an independent impact evaluation that will assess the impact of Durham Connects on: (1) child maltreatment; (2) mother and infant health and health care utilization, (3) parenting, parent-child relationship quality, and child care utilization and development and parenting; (4) family service receipt and connection to community resources; and (5) early childhood education. The aforementioned outcomes of the proposed project are all important and innovative contributions that are necessary to advance the field of early childhood programs. By determining whether previously identified positive program impacts (described below) can be replicated in an independent randomized controlled trial (RCT), the project seeks to reveal opportunities for transformational change to fundamentally improve population-level health and well-being outcomes for infants and families.

Background & Significance

- Should support the scientific aims of the research

Federal support for evidence based home visiting programs has accelerated in recent years, with $1.5 billion provided through the Maternal, Infant, and Early Childhood Home Visiting program (MIECHV). Although multiple targeted home visiting programs have demonstrated efficacy in improving child and family well-being through rigorous RCTs (e.g., Nurse-Family Partnership, Health Families America), scaling up early interventions can degrade quality by as much as to 50% (Welsh et al., 2010); importantly, no home visiting program has ever been scaled up to successfully improve child and family well-being for an entire community. The *DC* program was designed to address limitations to targeted programs that prohibit scaling for population-level impact. It is brief and inexpensive ($700 per family) so that communities can afford its costs. Further, it was designed for population-level implementation from the start, so no scaling is required. Results from an 18-month RCT of *DC* show that the program can reach a high percentage of the community (69% of eligible families completed the program) with high fidelity of implementation (Dodge et al, 2014).

Results from an independent impact evaluation at infant ages 6- and 12-months suggest multiple positive program effects on child and family well-being. Specifically, interviews with a random, representative subsample of 549 families at infant age 6-months suggest that random assignment to the *DC* program (vs. services as usual) was associated with: more connections to community resources within the past 3 months; utilization of higher quality child care when child care was used; greater mother-infant relationship and home environment quality as rated by blinded interviewers; lower rates of maternal anxiety and depression; and reduced utilization of infant emergency medical care (Dodge et al., 2014). Further, results from analyses of administrative hospital records indicate that infants randomly assigned to *DC* utilized 57% less emergency department care (ER visits + hospital overnights) through age 6-months, and 50% less emergency department care through age 12-months. Importantly, these results were observed for multiple demographic subgroups, suggesting universal needs and impacts for families (Dodge et al., 2013). Benefit-cost analyses based on implementation costs and average cost of child emergency care suggest $3.02 in emergency health care savings for each $1.00 spent on *DC* within the first year. For a community of the size of Durham, NC, with an average of 3,187 resident births per year and intervention cost of $700 per birth, an investment of $2,230,900 for the *DC* Program yields a community-wide emergency health care cost savings of $6,737,318 (Dodge et al, 2014).

Overall, results from the initial *DC* RCT suggest universal, postpartum nurse home visiting can obtain significant positive effects for family well-being and infant health outcomes through age 12-months. While highly promising, these results have yet to be replicated, a critical step for establishing an evidence-based home visiting model (Avellar et al., 2013). *The current proposal is significant because it uses an RCT to evaluate the replication of a novel, universal home visiting program with promising evidence suggesting population-level impact on health, well-being, and health care costs.*

Design & Procedures

- Describe the study, providing detail regarding the study intervention (drug, device, physical procedures, manipulation of the subject or the subject's environment, etc.). Discuss justifications for placebo control, discontinuation or delay of standard therapies, and washout periods if applicable. Identify procedures, tests and interventions performed exclusively for research purposes or more frequently than standard of care. Include alternative therapies, concurrent therapies discontinued per protocol, risk benefit ratio, and use of tissue /specimens. Discuss monitoring during washout periods if applicable. Include brief description of follow-up, if any.

Participants will include families of all resident Durham County births from January 1, 2014 – June 30, 2014 at Duke and Durham Regional hospitals. All families will be identified and recruited from publicly available short form birth records. Research interviews will take place in when infants are between 4-8 months old. The majority of research interviews will occur in participant homes; however, if the participant would prefer that the interview be conducted elsewhere, the interview may be conducted at the Center for Child and Family Policy at Duke, or at a public location, such as a public library. For any interview conducted outside of the participant's home, interviews will be conducted in a private setting, such as a conference room or private reading room in order to protect participant confidentiality. Procedures will include an introduction to the study, informed consent procedures, and a brief demographic and psychosocial interview. The interview (approximately 20-30 minutes long) will consist of a maternal interview about parenting and parent-child relationship quality, mother and child health and health care utilization, family service receipt, and maternal mental health, as well as staff observations of the home environment. For monolingual Spanish mothers and mothers who prefer Spanish language interviews,

procedures will be conducted in Spanish. Research interviewers will not be provided with information regarding family participation (or not) in the Durham Connects program to avoid the introduction of bias in data collection due to knowledge of program participation. The interview responses will be completed using paper forms in the subject's home and then entered into a Qualtrics database at the Center for Child and Family Policy.

All mothers will also be asked for consent to access other community service records in which the family might have participated, including Durham Connects records, to confirm any participation. We anticipate that about half of the participants will have participated in the Durham Connects program and about a quarter will have participated in other family community services. Analyses will examine child and family outcomes and service receipt as a function of previous service participation (e.g., Durham Connects, other services).

Participants will also be asked to sign several individual consent forms for community service agencies that require internal forms to be filled out in order to provide any records that a family might accumulate. With these records, research staff would like to complete child health assessments by reviewing any medical records, community agency utilization, and other health indicators/milestones that can be gathered from these records. Consent for release of this information from medical/service providers will be obtained during the interview with the mothers. Record collection and chart audits will occur in the practices/offices.

Additionally, following written parental consent, we will review and analyze records from the North Carolina Division of Social Services (DSS), Department of Health and Human Services and the Department of Public Instruction/Public Schools as well as other county hospital records and health care providers. Every 12 months, NC DSS records of alleged and substantiated child abuse and neglect are transferred to the Center for Child and Family Policy (Durham Family Initiative Part I: Archival Data Review, Pro00010890). Our staff will review these records to search (using a SAS matching program and a manual review of the data) for any indication that the infant/target child was an alleged or substantiated victims of abuse or neglect or had maltreatment-related diagnoses up to age 12. Additionally, local county hospital admission and emergency department records (including records from all Duke Medical System Hospitals), as well as other health care provider records, provided to the Center will be reviewed for rates of mother and infant healthcare utilization, as well as healthcare costs. DUMC records will be requested every 12 months via the Duke Health Technology Systems (DHTS) data request website. Mother and child data on child healthcare utilization, as well as healthcare costs will be examined through child age 12, including dates of admission and discharge, type of visit, hospital and physicians billing costs, and relevant family demographic information. Hospital admission and emergency department records will also be reviewed for ICD-9 diagnostic codes for children associated with possible maltreatment. Consent for release of this information will be obtained during the initial interview with the mothers when the child is 4-8 months of age.

Although research interviews will be limited to families with an infant between 4-8 months of age because the questionnaires are specific to that developmental period, administrative record data are collected retrospectively so infant age is not a threat to study validity for these data. Therefore, in the event that an eligible family that has previously "timed out" due to infant age contacts study staff (e.g., in response to a study flyer) and wishes to participate in the study, these families will be offered an opportunity to participate in the administrative record portion of the study only. **Study staff will not actively recruit families with an infant older than 8 months**.

Selection of Subjects

- List inclusion/exclusion criteria and how subjects will be identified.

We will use publicly available short-form birth records to identify eligible participants. The birth records include key information needed to contact eligible families, including parent names, child name, child date of birth, family address, parents' race/ethnicity, and hospital of birth. The research team will attempt to contact and recruit families of all resident Durham County births from January 1, 2014 – June 30, 2014 at Duke and Durham Regional hospitals. A total of 1800 participants will be enrolled. Because the study enrollment design includes all families that could be enrolled in the study sample *a priori*, families that decline participation will not be replaced.

Subject Recruitment and Compensation

- Describe recruitment procedures, including who will introduce the study to potential subjects. Describe how you will ensure that subject selection is equitable and all relevant demographic groups have access to study participation (per 45 CFR 46.111(a) (3)). Include information about approximately how many DUHS subjects will be recruited. If subjects are to be compensated, provide specific prorated amounts to be provided for expenses such as travel and/or lost wages, and/or for inducement to participate.

Using the contact information in the publicly-available birth records, recruitment will begin with an introductory information/recruitment letter mailed to identified families explaining the study, why we are contacting them, how we received their contact information, and how they can get in touch with us in case they have questions, comments or would like to decline to participate. The following recruitment steps will be used, in this order:

1. Identify potential subject and mail initial recruitment letter with return reply card.

2. Wait for 10 days to 2 weeks, then try to contact with phone. If telephone number supplied, but if no answer or not able to leave voice mail, then may proceed to step 3.

3. If no contact made as above (not able to leave voice mail or speak with someone), then may go to home ONLY once, and speak with potential subject and/or leave "in-person" recruitment letter. Visits should occur: 9am -6 pm only

4. Final step, may send "final" recruitment letter

If no response, then must STOP.

The study team will document times/methods/steps made to contact potential subjects. Contact should only be made at residence, not place of business. If a recruitment reply card is returned indicating that a potential participant will contact project staff, project staff will allow 2 weeks for contact. If the potential subject attempts no contact after 2 weeks, project staff will attempt to contact the potential subject following recruitment steps #2 - #4 described above. If direct contact is made by telephone or in-person and the participant indicates they are interested in participating in the study but does not follow-up with project staff after 1 week, project staff will attempt to contact the potential subject following recruitment steps #2 - #4. Interviewers will attempt to follow-up in this scenario by telephone whenever possible; in-person contact will only be made if no valid telephone number is available (*in this case, would you consider it reasonable to skip step #3?*). If direct contact is made and the response is neutral (e.g. individual wants time to think about it) and participant does not follow-up, a final recruitment letter will be mailed (i.e., Step #4 only).

The initial letter will include a reply card with an addressed, stamped envelope that the subject will be asked to return indicating their interest to participate or to not participate. A $10 gift card will be offered if the subject declines participation by one of the methods listed in the initial recruitment letter. The letter will also offer email and voicemail alternatives to communicate their decision. In order to best reach families, we will conduct searches on Lexus Nexus (paid service) to back match available addresses with telephone numbers. Only publically available information is used in this search, specifically participant name, address, and date of birth. In the event that information extracted from birth records is insufficient or inaccurate, we aim to use methods such as other publicly available social networking sites, such as Facebook or MySpace, as well as White Pages, Autotrack, and other methods of searching for people on publicly available networks. This information will be used to help determine residence outside of Durham County (a criteria that would make families ineligible to participate), **but we will not contact families directly via social media.**

To increase the likelihood of locating eligible participants, recruitment flyers will also be posted in locations throughout Durham County where families with young children frequently visit, such as doctors' offices, public parks and apartment or housing complexes. The flyer will identify the study, including eligibility criteria, and provide study contact information for potential participants. Appropriate permission will be obtained prior to posting flyers when required (e.g., posting at a doctor's office). Finally, project staff may also attend community meetings to describe the project to families that may be interested/eligible (e.g., a meeting of a homeowner's association or housing authority meeting). Any public presentations, however, will be limited to a description of the study and eligibility criteria – families will not be recruited or enrolled in a public meeting to protect participant confidentiality. Copies of the study recruitment letter will be left at these meetings so that interested families can obtain staff contact information.

Participants may be compensated up to $40 for participating in this study. For participating in the interview, participants will be given a $20 pre-paid gift card from either Wal-Mart or Target. Participants will also be offered a $10 a gift card for completing the consent to utilize Social Security or Medicaid numbers and the consent for child education records, respectively ($20 total).

Consent Process

- Complete the consent section in the iRIS Submission Form.

Subject's Capacity to Give Legally Effective Consent

- If subjects who do not have the capacity to give legally effective consent are included, describe how diminished capacity will be assessed. Will a periodic reassessment occur? If so, when? Will the subject be consented if the decisional capacity improves?

Cognitive capacity will be assessed through interactions and the interview itself, and potential participants with known or suspected cognitive impairment will not be recruited. Mental health needs are a part of the existing interview, and potential participants who present with acute and emergent needs will not be eligible for inclusion. Any participating mothers under 18 years of age will be informed of the study with a custodial parent/guardian present, and consent will be requested from both the parent and the teenaged mother.

Study Interventions

- If not already presented in #4 above, describe study-related treatment or use of an investigational drug or biologic (with dosages), or device, or use of another form of intervention (i.e., either physical procedures or manipulation of the subject or the subject's environment) for research purposes.

N/A

Risk/Benefit Assessment

- Include a thorough description of how risks and discomforts will be minimized (per 45 CFR 46.111(a) (1 and 2)). Consider physical, psychological, legal, economic and social risks as applicable. If vulnerable populations are to be included (such as children, pregnant women, prisoners or cognitively impaired adults), what special precautions will be used to minimize risks to these subjects? Also identify what available alternatives the person has if he/she chooses not to participate in the study. Describe the possible benefits to the subject. What is the importance of the knowledge expected to result from the research?

This study is expected to pose minimal risks to participating mothers and their children. The proposed procedures and measures have been used widely in developmental psychology research involving participants with no identified problems as well as those with very pronounced problems. All questionnaires included in this study have been previously implemented in the Prospective Evaluation of Infant Development (Pro00020974) with no adverse incidents. There are no physical risks to participants. Portions of the assessment measures may cause some temporary discomfort or uneasiness; however serious adverse effects are unlikely and have not been encountered in our work to date or, to our knowledge, in previous studies with these measures. As noted above, participants will be reminded that they are free to decline to answer any question, that they may stop answering or withdraw consent for sharing their administrative record data at any time without penalty, and that steps will be taken to ensure the confidentiality of their data. There is some possibility that participants may experience discomfort in answering interview questions and that the interview may identify emergent clinical needs (e.g., depression, anxiety). If a participant's responses indicate that referrals are appropriate (e.g., participant indicates distress that is impairing their functioning, participant expresses interest in seeking clinical help),

research staff will provide information on referrals for appropriate counseling and/or substance use treatment services.

One risk for participants is the potential loss of confidentiality. Great care will be taken to ensure that confidentiality is protected for all participants, as described in the Research Data Security Protocol (RDSP). Additionally, it is possible that observations and maternal reports to research assistants may reveal information regarding harm or serious risk of harm to children which would be subject to mandatory reporting requirements. Prior to each assessment session, participants will be informed about mandatory reporting and the risks of disclosure, and that they may choose to skip the items that cause them to feel uncomfortable. In an effort to minimize risk associated with reporting child maltreatment, research staff will discuss the issue with participants prior to making any report, except when it is deemed dangerous to the child or to the person filing the report to do so. We will permit participants to be involved in reporting the maltreatment themselves if they choose, which can reduce the stress that a participant may experience as a result of a report being made.

Information from the study may directly benefit the participants in the study by giving them the opportunity to reflect on their family's circumstances and experiences. In the longer run, findings are expected to advance the fields of preventive intervention and home visiting through the evaluation of community-wide programs that aim to deliver effective, personalized care.

In comparison to the anticipated benefits to participants and others, most of the risks to participants in this study are minimal, including only some discomfort and/or increased self-consciousness for mothers. More serious risks to participants, such as being reported for child maltreatment, are expected to occur infrequently. In sum, the minimal risks to participants are reasonable in relation to the importance of the knowledge that may reasonably be expected to result from the proposed study.

Costs to the Subject

- Describe and justify any costs that the subject will incur as a result of participation; ordinarily, subjects should not be expected to pay for research without receiving direct benefit.

Study participants will not incur any costs as a result of participation.

Data Analysis & Statistical Considerations

- Describe endpoints and power calculations. Provide a detailed description of how study data will be analyzed, including statistical methods used, and how ineligible subjects will be handled and which subjects will be included for analysis. Include planned sample size justification. Provide estimated time to target accrual and accrual rate. Describe interim analysis including plans to stop accrual during monitoring. Phase I studies, include dose escalation schema and criteria for dose escalation with definition of MTD and DLT.

Statistical analyses involved in this project include basic descriptive analyses and examination of child and family outcomes and service receipt as a function of previous service participation (e.g., *Durham Connects*, other services). The primary research question is whether the *Durham Connects* program eligibility results in increased utilization of community resources and better child and family outcomes (e.g., , fewer official reports of maltreatment, reduced utilization of emergency healthcare, enhanced parenting and child health and development outcomes) compared to families that were not eligible for the program. The second research is whether the *Durham Connects* program will have larger intervention effect sizes for higher-risk groups, including families with Medicaid or no health insurance, racial/ethnic minority families, single parent families, and infants with one or more birth risks (i.e., premature birth, low birth weight, or other birth complications).

Ordinary Least Squares (OLS) regression models will test hypotheses for continuous outcome variables; Multivariate logistic regression models will test hypotheses for dichotomous outcome variables; multivariate Poisson regression models will test hypotheses for count outcomes with non-normal distributions (i.e., maltreatment investigations and emergency department presentations; Coxe, West, & Aiken, 2009). Analyses will be conducted using a two-tailed "intent-to-treat" design that includes all intervention and control families without regard for intervention adherence or receipt. Moderation effects will be tested by generating interaction terms for demographic and risk variables. In the event of significant effects, in follow-up tests will be conducted using techniques described by Aiken & West (1991). Though concerted efforts will be made to maintain the maximum sample possible, some missing

observations are expected. Full information maximum likelihood (FIML) estimation will be applied to analytic models to address missing data, in order to reduce bias in the parameter estimates and to preserve statistical power.

DSS reports of child maltreatment are likely to occur at relatively low frequency, particularly given that reporting may greatly underestimate actual maltreatment rates. To maximize power, survival analyses will be used to examine group differences. Child and family outcomes with continuous scores and greater variability will also be examined. Using power calculation software, we found the study to be adequately powered to detect significant differences for our principal hypotheses.

Data & Safety Monitoring

- Summarize safety concerns, and describe the methods to monitor research subjects and their data to ensure their safety, including who will monitor the data, and the frequency of such monitoring. If a data monitoring committee will be used, describe its operation, including stopping rules and frequency of review, and if it is independent of the sponsor (per 45 CFR 46.111(a) (6)).

Data security will be carefully monitored in the data collection, processing, and analysis stages (see RDSP for a more comprehensive description of policies and procedures). The data will be stored on a secure network server managed by the Duke University Office of Information Technology (Richard Biever, Chief Information Officer, 919-684-8121), which requires a Virtual Private Network (VPN) connection to access. The VPN connection requires a Duke University NetID and NetID password. Folder access will be further monitored and restricted by members of the Sanford School of Public Policy Office of Information Technology, such that only authorized users approved by the study PI will have access to the file server. Original data will be kept in locked compartments separate from documentation and access information. No electronic data will be stored outside of the secure network (e.g., on removable storage devices); all data analyses will be conducted in a virtual computing environment on a server located within the secure network that does not have an outside Internet connection (i.e., no email, web browser).; printouts derived from data analysis will be stored in a locked compartment when not in use and destroyed when no longer needed. All electronic and paper files that contain identifying information will be destroyed at the conclusion of the research. Published data from the proposed study will be in the form of group-level data and will not permit identification of individuals.

Privacy, Data Storage & Confidentiality

- Complete the Privacy and Confidentiality section of the iRIS submission form.

Describe Role of External Personnel:

N/A

Study Scope

Does the subject population contain >50% malignant hematology or oncology patients, or their caregivers?

○ Yes ◉ No

Are you using a drug, biologic, food, or dietary supplement in this study?

○ Yes ◉ No

Are you using a medical device, an algorithm (whether computer based or not), an in vitro diagnostic test, or using samples to look for biomarkers in this study?

( ) Yes (•) No

Does this study employ magnetic resonance, including imaging (MRI), spectroscopy (MRS), angiography (MRA) or elastography (MRE) beyond the standard of care?

( ) Yes (•) No

Does this study specify or require the performance of diagnostic procedures using ionizing radiation (x-rays, DEXA, CT scans, nuclear medicine scans, etc.) that are beyond the standard of care?

( ) Yes (•) No

Does this study specify or require the performance of therapeutic procedures using ionizing radiation (accelerator, brachytherapy or systemic radionuclide therapy) that are beyond the standard of care?

( ) Yes (•) No

Will the participant be subjected to increased or decreased ambient pressure?

( ) Yes (•) No

Do you plan to recruit subjects from Duke Regional Hospital (DRH)?

( ) Yes (•) No

Do you plan to recruit subjects from Duke Raleigh Hospital (DRAH)?

( ) Yes (•) No

Does this study utilize the Duke Early Phase Clinical Research Unit (DEPCRU)?

( ) Yes (•) No

Are you using the Duke logo in any advertisements?

( ) Yes (•) No

Is this study retrospective, prospective, or both?

"Retrospective" means that data or samples already in existence (collected prior to the study submission) will be used. "Prospective" means there will be data or samples collected in this study for research purposes.

( ) Retrospective
(•) Prospective
( ) Retrospective and Prospective

Does this protocol include any research using botulinum toxin, including the FDA-approved clinical product (Botox)?

( ) Yes (•) No

Does this protocol involve the administration of any of the following materials to humans?

•Any viral vector or plasmid
•Any cells that have been modified by a viral vector
•Any other genetically-modified cells
•Any genetically-modified virus, bacterium, or other agent
•Any other recombinant or synthetic nucleic acid

○ Yes ◉ No

Subject Population Groups and Enrollment

Population Groups (Select targeted population groups only):

Note:

- If Minors are included, the study will be routed to the Department of Pediatrics for Pediatric Risk Assessment.
- Students and Employees over whom Key Personnel have a supervisory role may not be enrolled in this study.
- Healthy Controls must be given a Notice of Privacy Practices.

- [x] Adults
- [ ] Minors who are Wards of State
- [x] Minors
- [ ] Duke Patients
- [ ] Pregnant Women
- [ ] Fetuses
- [ ] Prisoners
- [ ] Adults incapable of giving consent
- [ ] Adults with diminished capacity
- [ ] Handicapped subjects
- [ ] Students
- [ ] Employees
- [ ] Healthy Controls
- [ ] Deceased subjects
- [ ] Blanket Protocol

This study will be routed to the Department of Pediatrics Chair for Pediatric Risk Assessment.

Please select any population groups excluded from participation in this study:

- [ ] Pregnant women

Maximum number of subjects to be consented at Duke:

Enter a single number. If you anticipate consenting a range of subjects, enter the **upper** limit of the range. The number should represent the maximum number of subjects for the life of the study.

1800

Maximum number of subjects to be consented at all sites:

Enter a single number. If you anticipate consenting a range of subjects, enter the **upper** limit of the range. The number should represent the maximum number of subjects for the life of the study.

1800

Subject Procedures and Costs

Biobank - Does this study involve the collection, use, tracking, banking (storage) or distribution of human biological specimens?

Human biological specimens include blood or its components, healthy or diseased tissue, bodily fluids, DNA/RNA or human stem cells.

○ Yes ◉ No

Procedures

Check all the apply:

- [ ] Genetic Testing
- [ ] Gene Transfer
- [ ] DNA Banking
- [ ] Testing for Reportable Infectious Diseases
- [ ] Human Cell Banking
- [ ] *Use of Human Embryonic Stem Cells
- [ ] *Use of Human-induced Pluripotent Stem Cells
- [ ] *Use of Other Cells Derived from Human Embryos
- [ ] *Use of Human/Animal Chimeric Cells
- [ ] *Specialized Cell Populations for Cell Therapy
- [ ] Use of Human Tissue
- [ ] Use of Bodily Fluids
- [ ] Use of Blood (or its components)
- [x] Not Applicable

Will blood be drawn in this study for research purposes?

○ Yes ◉ No

Will the Operating Room be used in this study?

Include only research time, not clinical care time.

○ Yes ◉ No

Will there be extra costs to subjects or insurance as a result of the research (e.g. tests, hospitalization)?

○ Yes ◉ No

Will there be Subject Compensation?

◉ Yes ○ No

Compensation for Travel / Lost Income (in USD):

40

Other Subject Compensation:

Participant receiving a follow-up recruitment letter that contact our interviewers to let them know they are not interested in participating will receive a $10 gift card.

Subject Recruitment Materials

For each document to be reviewed, use the table below to provide the following information:

Attach a copy of each advertisement that you will be using with this study in the Initial Submission Packet. If any Ad will have multiple wording variations, attach a copy of each version of the Ad.
All materials that will be used to advertise the study in order to recruit subjects must be approved by the IRB.

Types of subject recruitment materials include, but are not limited to, the following:

Direct Advertising
Posters
Billboards
Flyers
Brochures

Media Advertising
Newspaper Ads
Magazine Ads
Radio Ads
TV commericals / Video
Internet website
Social Media

Other Types of Advertising
Newsletter
Email
Postcards / Letters

(Note: Doctor-to-Doctor letters do not require IRB approval)

Document name	Material category	Location material displayed	Has this material previously been approved by the IRB?
Recruitment Letter-English	○ Billboard / Flyer / Poster<br>○ Brochure<br>○ Internet website / Email<br>◉ Letter / Postcard<br>○ Phonescript<br>○ Radio<br>○ Television / Video<br>○ Newsletter / Newspaper / Magazine<br>○ Other	Please be specific. For example, "Duke" would not be an appropriate location. "Duke Hospital Television" would be an appropriate response.<br><br>This letter will be mailed to all potential participants to inform them that they have been selected to participant in this study.	○ Yes ◉ No
Recruitment Letter-Spanish	○ Billboard / Flyer / Poster<br>○ Brochure<br>○ Internet website / Email<br>◉ Letter / Postcard<br>○ Phonescript<br>○ Radio<br>○ Television / Video<br>○ Newsletter / Newspaper / Magazine<br>○ Other	Please be specific. For example, "Duke" would not be an appropriate location. "Duke Hospital Television" would be an appropriate response.<br><br>This letter will be mailed to all potential participants to inform them that they have been selected to participant in this study.	○ Yes ◉ No
Recruitment Script-English	○ Billboard / Flyer / Poster<br>○ Brochure<br>○ Internet website /	Please be specific. For example, "Duke" would not be an appropriate	○ Yes ◉ No

	Email<br>○ Letter / Postcard<br>◉ Phonescript<br>○ Radio<br>○ Television / Video<br>○ Newsletter / Newspaper / Magazine<br>○ Other	location. "Duke Hospital Television" would be an appropriate response.<br><br>This script will be read to all potential participants when contacted by telephone regarding participation in the study	
Recruitment Script-Spanish	○ Billboard / Flyer / Poster<br>○ Brochure<br>○ Internet website / Email<br>○ Letter / Postcard<br>◉ Phonescript<br>○ Radio<br>○ Television / Video<br>○ Newsletter / Newspaper / Magazine<br>○ Other	Please be specific. For example, "Duke" would not be an appropriate location. "Duke Hospital Television" would be an appropriate response.<br><br>This script will be read to all potential participants when contacted by telephone regarding participation in the study	○ Yes ◉ No
Follow-Up Recruitment Letter - English	○ Billboard / Flyer / Poster<br>○ Brochure<br>○ Internet website / Email<br>◉ Letter / Postcard<br>○ Phonescript<br>○ Radio<br>○ Television / Video<br>○ Newsletter / Newspaper / Magazine<br>○ Other	Please be specific. For example, "Duke" would not be an appropriate location. "Duke Hospital Television" would be an appropriate response.<br><br>Follow-up recruitment letter will be mailed to only those families that do not respond to our initial recruitment efforts.	○ Yes ◉ No
Follow-Up Recruitment Letter - Spanish	○ Billboard / Flyer / Poster<br>○ Brochure<br>○ Internet website / Email<br>◉ Letter / Postcard<br>○ Phonescript<br>○ Radio<br>○ Television / Video<br>○ Newsletter / Newspaper / Magazine<br>○ Other	Please be specific. For example, "Duke" would not be an appropriate location. "Duke Hospital Television" would be an appropriate response.<br><br>Follow-up recruitment letter will be mailed to only those families that do not respond to our initial recruitment efforts.	○ Yes ◉ No
Recruitment Reply Card - English	○ Billboard / Flyer / Poster<br>○ Brochure<br>○ Internet website / Email<br>◉ Letter / Postcard	Please be specific. For example, "Duke" would not be an appropriate location. "Duke Hospital Television" would be an appropriate response.	○ Yes ◉ No

	○ Phonescript<br>○ Radio<br>○ Television / Video<br>○ Newsletter / Newspaper / Magazine<br>○ Other	Postcard and addresses, stamped envelope will be included with recruitment letter in initial recruitment mailing to families.	
Recruitment Reply Card - Spanish	○ Billboard / Flyer / Poster<br>○ Brochure<br>○ Internet website / Email<br>◉ Letter / Postcard<br>○ Phonescript<br>○ Radio<br>○ Television / Video<br>○ Newsletter / Newspaper / Magazine<br>○ Other	Please be specific. For example, "Duke" would not be an appropriate location. "Duke Hospital Television" would be an appropriate response.<br><br>Postcard and addresses, stamped envelope will be included with recruitment letter in initial recruitment mailing to families.	○ Yes ◉ No
Recruitment Flyer - English	○ Billboard / Flyer / Poster<br>○ Brochure<br>○ Internet website / Email<br>○ Letter / Postcard<br>○ Phonescript<br>○ Radio<br>○ Television / Video<br>○ Newsletter / Newspaper / Magazine<br>○ Other	Please be specific. For example, "Duke" would not be an appropriate location. "Duke Hospital Television" would be an appropriate response.<br><br>Copies of this flyer will be posted in doctors' offices and public spaces, such as parks or libraries. When required, appropriate permissions will be obtained prior to posting flyer (e.g., approval from a doctor's office or clinic).	○ Yes ◉ No
Recruitment Flyer - Spanish	○ Billboard / Flyer / Poster<br>○ Brochure<br>○ Internet website / Email<br>○ Letter / Postcard<br>○ Phonescript<br>○ Radio<br>○ Television / Video<br>○ Newsletter / Newspaper / Magazine<br>○ Other	Please be specific. For example, "Duke" would not be an appropriate location. "Duke Hospital Television" would be an appropriate response.<br><br>Copies of this flyer will be posted in doctors' offices and public spaces, such as parks or libraries. When required, appropriate permissions will be obtained prior to posting flyer (e.g.,	○ Yes ◉ No

		approval from a doctor's office or clinic).	
In-Person Recruitment Letter - English	◯ Billboard / Flyer / Poster<br>◯ Brochure<br>◯ Internet website / Email<br>◉ Letter / Postcard<br>◯ Phonescript<br>◯ Radio<br>◯ Television / Video<br>◯ Newsletter / Newspaper / Magazine<br>◯ Other	Please be specific. For example, "Duke" would not be an appropriate location. "Duke Hospital Television" would be an appropriate response.<br><br>This letter will be provided to potential participants during one in-person recruitment to inform them that they have been selected to participant in this study.	◯ Yes ◉ No
In-Person Recruitment Letter - Spanish	◯ Billboard / Flyer / Poster<br>◯ Brochure<br>◯ Internet website / Email<br>◉ Letter / Postcard<br>◯ Phonescript<br>◯ Radio<br>◯ Television / Video<br>◯ Newsletter / Newspaper / Magazine<br>◯ Other	Please be specific. For example, "Duke" would not be an appropriate location. "Duke Hospital Television" would be an appropriate response.<br><br>This letter will be provided to potential participants during one in-person recruitment to inform them that they have been selected to participant in this study.	◯ Yes ◉ No

Consent Process

Attach draft consent forms in the Initial Review Submission Packet.

Consent forms must be MS Word documents and follow the specific format outlined by the IRB. **Click here** to download a copy of the consent form template.

Note: Please do not edit the section of the footer that contains the Protocol ID, Continuing Review and Reference Date fields. Those fields will be used to stamp the final consent form when it is approved by the IRB. If you want to add an internal version date, please put it in the header.

Who will conduct the consent process with prospective participants?

Give the person's role in this study (PI, Study Coordinator, etc.):

A team of experienced research aides will be employed to conduct the consenting process with the described sample. These research aides have prior experience consenting participants on similar protocols previously approved by the Duke Medicine IRB (Duke Medicine IRB #s Pro00010890, Pro00011136).

Who will provide consent or permission?

(Select all that apply):

- [x] Participant
- [x] Parent(s) or Legal Guardian(s)
- [ ] Legally Authorized Representative (LAR)

How much time will the prospective participant (or legally authorized representative) have between being approached about participating in the study and needing to decide whether or not to participate?

If you are not giving the person overnight to consider whether or not to participate, please justify.

An letter describing the project and inviting the prospective participant to participate in the study will be mailed 2 weeks prior to contacting the participant in person or by telephone. This letter will describe the purpose of the project and provides the contact information for the project coordinator (Jeff Quinn) should the participant have any questions or concerns.

When the consenting process occurs, the research interviewer will review the consent form in detail and provide the participant with ample opportunity to ask any questions or express any concerns prior to providing informed consent. All participants will be informed that they can choose not to participate in any portion of this protocol or withdraw from the study at any time without penalty.

Where will the consent process occur?

The consenting process will occur in the a setting chosen by the participant. This will generally include the participant's place of residence, but may also include the Center for Child and Family Policy at Duke University or a public location, such as a public library.

What steps will be taken in that location to protect the privacy of the prospective participant?

Research interviewers will confirm that interviews completed at the participant's residence are conducted at a time that is convenient for the participant, and that there are no concerns about privacy prior to obtaining informed consent. If the interview is completed at the Center for Child and Family Policy, informed consent and interviewing will be completed in a private conference room within the suite. If the interview is completed in a public location, such as a public library, the informed consent and interviewing process will be completed in a private location, such as a conference room or study room.

How much time will be allocated for conducting the initial consent discussion, including presenting the information in the consent document and answering questions, with each prospective participant?

Between 15 and 30 minutes will be allocated for the initial consent discussion for each participant.

What arrangements will be in place for answering participant questions before and after the consent is signed?

Trained research aides well-versed in the projects consenting procedures will be able to answer most questions during contact. For any unanswered questions, the study coordinator's contact information is provided on the consenting form.

Describe the steps taken to minimize the possibility of coercion or undue influence.

All participants will be informed that they can choose not to participate in any portion of this protocol or withdraw from the study at any time without penalty. Participants are compensated for their time, but the compensation amount ($40) is appropriate for the amount of time required to complete the consenting and interview process (30-40 min.).

What provisions will be in place to obtain consent from participants who do not read, are blind or who do not read/understand English?

In any instance where the participant or research aide has concerns about the participant's ability to read or understand the consent form, the research aide will read the consent form aloud to the participant and explain any portions that may be unclear. Additionally, half of the research aides for this project are bilingual (English and Spanish language) to accommodate the Hispanic population participating in this study.

Do you plan to obtain written consent for the conduct of research?

(•) Yes ( ) No

Protected Health Information (PHI)

Indicate how you intend to use potential subjects' Protected Health Information (PHI):

( ) I will review, but not record, PHI prior to consent.
( ) I will record PHI prior to consent.
(•) I do not intend to use PHI prior to consent.
( ) I will record PHI without consent. (decedent research, database repository, chart review)

Privacy and Confidentiality

Explain how you will ensure that the subject's privacy will be protected:

Consider privacy interests regarding time and place where subjects provide information, the nature of the information they provide, and the type of experience they will be asked to participate in during the research.

Considerable care will be given to avoid inadvertent disclosure of confidential information about the study participants. All participants will be identified by random, confidential ID numbers. A spreadsheet linking respondents, ID numbers, addresses, and phone numbers will be kept on a secure server maintained by Duke University OIT that requires a virtual private network (VPN) to access (VPN requires valid NetID and NetID password). Access to this spreadsheet will be restricted to essential project staff listed on this IRB protocol. Hard copies of project consent forms (which contain identifying data) will be kept in a locked cabinet within the project offices to assure confidentiality. Staff members who are directly involved in the recruitment and data processing will have access to the identified data regarding the research interviews. All research staff will be required to sign a pledge of confidentiality that acknowledges each project member's responsibility to guard against unauthorized use or disclosure of any identifiable information about the participants. Interview data will be collected via computers or hard copies. Hard copies of interview data will be stored in locked file cabinets in a locked project office separate from signed consent forms. Published data from the proposed study will be in the form of group-level data and will not permit identification of individual project participant.

In addition to the interview data, the Center for Child and Family Policy will also be the recipients of outside databases such as DSS and hospital records. As noted above, our center has already been granted permission to access and analyze DSS records between 1997 and 2012 by the North Carolina and Durham County Departments of Social Services and by the DUMC IRB. An electronic copy of the identified data will be accessible to a single data manager and the manager's assistant, both well-trained in confidentiality procedures and ethics. These individuals will be responsible for reviewing administrative data and matching up cases with identifying information for all those who have consented to have their DSS and hospital records followed. Once the data are matched and appropriately linked to the current study data, identifiers other than the study number will be removed, and the data will be shared with the statistician and investigators for analysis via a file on a secure server. The database with DSS and hospital data attached will remain in separate files on the secure server, accessible only to the data manager and the assistant. Identified data will thus never be viewed by other study staff (e.g., statisticians or investigators).

Describe how research data will be stored and secured to ensure confidentiality:

How will the research records and data be protected against inappropriate use or disclosure, or malicious or accidental loss or destruction? Records and data include, for example, informed consent documents, case report forms or study flow sheets, survey instruments, database or spreadsheets, screening logs or telephone eligibility sheets, web based information gathering tools, audio/video/photo recordings of subjects, labeled specimens, data about subjects, and subject identifiers such as social security number.

All project data will be stored in locked cabinets in secured project offices. Electronic data gathered in home visits and pediatric offices will be on password-protected and encrypted on laptops. Padlocks will be secured on the laptop carry cases when the laptops are not in use. All data will be removed from the laptops within 24 hours and uploaded and backed up to a secure network server with password protection.

Data security will be carefully monitored in the data collection, processing, and analysis stages. All data will be stored on a secure network server maintained by Duke University OIT (Richard Biever, Chief Information Officer, 919-684-8121) that can only be accessed by project staff listed on this IRB. Access to data folders on the secure server is strictly controlled by the Office of Information Technology (OIT) staff at the Duke School of Public Policy, and requires a virtual private network (VPN) connection (requiring a valid NetID and NetID password). OIT staff will only provide server access to approved project staff after receiving written authorization from the project PI (Ken Dodge). No other individuals will be permitted access to the server. Paper records containing original data will be kept in locked compartments in a locked office separate from documentation and access information (i.e., consent forms and participant contact information). Electronic datasets will never be stored outside of the secure network environment (e.g, on a temporary storage drive); all data analyses will be conducted in a virtual computing environment on a server located within the secure network that does not have an outside Internet connection (i.e., no email, web browser). Any printouts derived from data analysis will be stored in a locked compartment when not in use and destroyed when no longer needed. All electronic and paper files that contain identifying information will be destroyed at the conclusion of the research.

Application Questions Complete

Please click Save & Continue to proceed to the Initial Submission Packet.

The Initial Submission Packet is a short form filled out after the protocol application has been completed. This is an area to attach protocol-related documents, consent forms, and review the application.

DukeHealth
Institutional Review Board

DUHS IRB NOTIFICATION OF CONTINUING REVIEW APPROVAL

Protocol ID: Pro00052735
Reference ID: 300186
Principal Investigator: Kenneth Dodge
Protocol Title: Impact Evaluation of a Durham Connects RCT Replication
Sponsor/Funding Source(s): National Institute of Child Health and Human Development
Federal Funding Agency ID:
Date of Declared Concordance with federally funded grant, if applicable: N/A

The Duke University Health System Institutional Review Board for Clinical Investigations has conducted the following activity on the study cited above:
Activity: Continuing Review
Review Type: Expedited
Review Date: 2/20/2019
Issue Date: 2/20/2019
Anniversary Date: 3/12/2019
Expiration Date: 3/12/2020

DUHS IRB approval encompasses the following specific components of the study:
Protocol, version/date:
DUHS IRB Application version: 1.2
Consent form reference date: closed
Investigator Brochure, version/date:
Pediatric Risk Category: 1
Other:

The DUHS IRB has determined the specific components above to be in compliance with all applicable Health Insurance Portability and Accountability Act ("HIPAA") regulations.

This study expires at 12 AM on the Expiration Date cited above. At that time, all study activity must cease. If you wish to continue specific study activities directly related to subject safety, you must immediately email Jody Power at jody.power@duke.edu or call the IRB Office at 919-668-5111 and follow the instructions to reach the IRB Chair on call. Continuing review submissions (renewals) must be received by the DUHS IRB office 60 to 45 days prior to the Expiration Date.

No change to the protocol, consent form or other approved document may be implemented without first obtaining IRB approval for the change. Any proposed change must be submitted as an amendment. If necessary in a life-threatening situation, where time does not permit your prior consultation with the IRB, you may act contrary to the protocol if the action is in the best interest of the subject. You must notify the IRB of your action within five (5) working days of the event.

The Duke University Health System Institutional Review Board for Clinical Investigations (DUHS IRB), is duly constituted, fulfilling all requirements for diversity, and has written procedures for initial and continuing review of human research protocols. The DUHS IRB complies with all U.S. regulatory requirements related to the protection of human research participants. Specifically, the DUHS IRB complies with 45CFR46, 21CFR50, 21CFR56, 21CFR312, 21CFR812, and 45CFR164.508-514. In addition, the DUHS IRB complies with the Guidelines of the International Conference on Harmonization to the extent required by the U. S. Food and Drug Administration.

DUHS Institutional Review Board
2424 Erwin Rd | Suite 405 | Durham, NC | 919.668.5111
Federalwide Assurance No: FWA 00009025

Statistical Analysis Plan (SAP)

DURHAM CONNECTS RCT II EVALUATION

Principal Investigator	Kenneth A. Dodge, PhD<br>Pritzker Professor of Early Learning Policy Studies<br>Sanford School of Public Policy<br>Professor of Psychology and Neuroscience<br>Duke University<br>Durham, NC
Protocol identification number	Pro00052735 (Impact Evaluation of a Durham Connects RCT Replication; Approved 02/19/2014)
ClinicalTrials.gov identifier	NCT01843036
Authors	Yu Bai, PhD<br>Statistician III<br>Center for Child and Family Policy<br>Duke University<br>Durham, NC<br><br>W. Benjamin Goodman, PhD<br>Research Scientist<br>Center for Child and Family Policy<br>Duke University<br>Durham, NC
Version	1.1

SIGNATURE PAGE

Principal Investigator

______________________________	____________________
Kenneth A. Dodge	Date

Authors

______________________________	____________________
Yu Bai	Date
______________________________	____________________
W. Benjamin Goodman	Date

Abbreviations

CPS	Child Protective Services
RCT	Randomized Controlled Trial

Table of Contents

ABBREVIATIONS 3

1. INTRODUCTION 5

2. STUDY DESIGN 5

2.1 SAMPLE SIZE CALCULATION 7

3. AIMS AND OBJECTIVES 7

4. OUTCOMES 7

4.1 PRIMARY OUTCOME 7

4.2 SECONDARY OUTCOMES 7

5. POPULATIONS AND SUBGROUPS TO BE ANALYZED 8

5.1 POPULATIONS 8

5.2 SUBGROUPS 9

6. ANALYSES 9

6.1 PRIMARY OUTCOME 9

6.2 SECONDARY OUTCOMES 10

7. MISSING DATA 10

1. Introduction

The aim of the current study is to test random assignment to the Durham Connects (now called Family Connects) community-wide newborn nurse home visiting program on: (1) child maltreatment assessments and substantiations, (2) mother and infant health and health care utilization, (3) parenting and parent-child relationship quality, and (4) family connections to community resources.

This statistical analysis plan (SAP) will give more detailed descriptions of the outcomes in the study and the corresponding analyses.

2. Study Design

Study subjects were originally to include all resident Durham County births from January 1, 2014 – June 30, 2014 at two county birthing hospitals (Duke University Hospital and Durham Regional Hospital). However, Durham Regional births were subsequently excluded from the trial due to an unanticipated change in hospital policies at Durham Regional prohibited patient contact with non-hospital employees (which invalidated the intervention implementation design for that hospital). The final study design includes all resident Durham County births from January 1, 2014 – June 30, 2014 at Duke University Hospital.

The study is a two-armed, parallel-design RCT. Families of infants born during the RCT enrollment period were randomized to be eligible to receive the Durham Connects home visiting intervention or to the control group. Families were randomized *a priori* to one of the two intervention groups based on infant birth date: 1) *odd birth date* families were assigned to receive Durham Connects; program staff attempted to engage all of these families and schedule a home visit; 2) *even birth date* families were not offered Durham Connects but received other community services as usual and served as the control group. Although differing from traditional randomization procedures in clinical trials, whereby individuals are randomized after providing informed consent, the *a priori* randomization procedure utilized in the current trial was necessary to examine program implementation and impact within the full community population (not only those families willing to participate in a randomized trial). This approach allowed for inclusion of all eligible families (i.e., families living in Durham County giving birth at Duke University Hospital) with experimental rigor, and without exception, but with ethical care for privacy.

To examine impact of assignment to intervention, a separate research evaluation design was implemented independently by a team unaffiliated with the intervention, beginning at age 6 months. Using short-form birth records, research assistants blind to experimental condition tried

to find all still-resident RCT families to solicit consent to participate in a research study of infant development. The study design is presented in Figure 1 below.

Figure 1. *CONSORT 2010 Flow Diagram for Family Connects RCT Implementation*

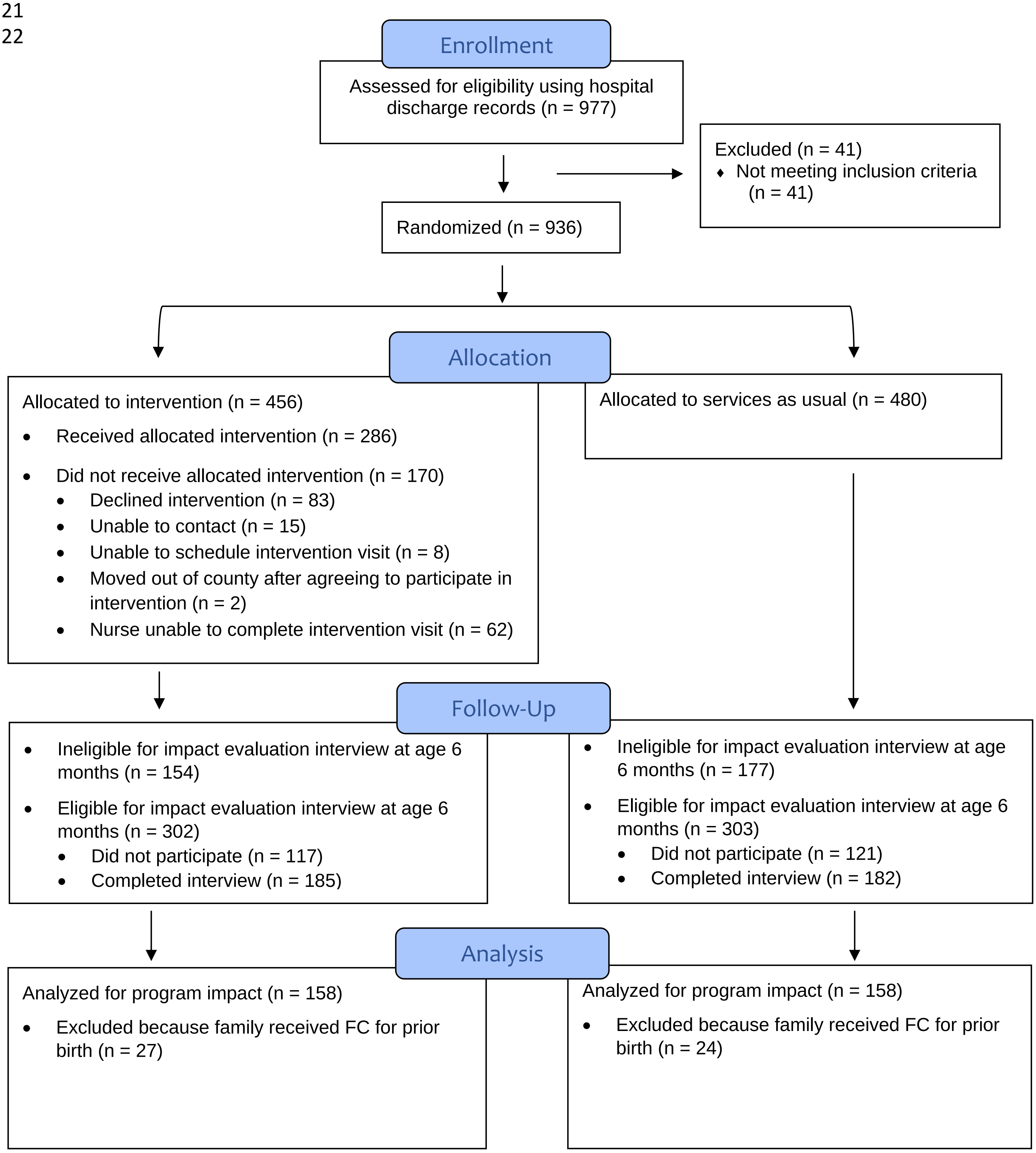

2.1 Sample Size Calculation

YU - ADD POWER ANLYSES HERE

3. Aims and Objectives

To examine whether random assignment to receive a brief, postpartum nurse home visiting program predicts (1) reductions in child maltreatment assessments and substantiations, (2) increases in mother and infant health and decreases in mother and infant emergency medical care utilization, (3) higher quality parenting behaviors and parent-child relationship quality, and (4) increased family connections to community resources.

4. Outcomes

This section will present the outcomes investigated to answer the study aims and objectives. The analyses are described in Section 6.

4.1 Primary Outcome

1. **Child maltreatment assessments and substantiations**. This outcome will be measured using CPS administrative records from the North Carolina Division of Social Services. Records will be collected beginning at birth and continue through child age 12 years.

4.2 Secondary Outcomes

1. **Child emergency room (ER) presentation rates**. This outcome will be measured using Duke University Health System administrative records of all outpatient emergency room presentations for the study child. Records will be collected beginning at birth and continue through child age 12 years.

2. **Child hospital overnight stays**. This outcome will be measured using Duke University Health System administrative records of all inpatient nights spent in hospital for the study child. Records will be collected beginning at birth and continue through child age 12 years.

3. **Mother emergency room (ER) presentation rates**. This outcome will be measured using Duke University Health System administrative records of all outpatient emergency room presentations for the study mother. Records will be collected beginning at birth of the study child and continue through child age 12 years.

4. **Mother hospital overnight stays**. This outcome will be measured using Duke University Health System administrative records of all inpatient nights spent in hospital for the study mother. Records will be collected beginning at birth of the study child and continue through child age 12 years.

5. **Mother postnatal well-care compliance.** This outcome will be measured based on mother self-report of completing (or not) her 6-week postpartum health exam. This outcome will be measured during an in-home interview when the study child is approximately 6 months old.

6. **Child postnatal well-care compliance.** This outcome will be measured based on mother self-report of having taken (or not) her child for a pediatric well-child exam within the past month. This outcome will be measured during an in-home interview when the study child is approximately 6 months old.

7. **Mother mental health.** This outcome will be measured based on mother self-report on two brief questionnaires: the Edinburgh Postnatal Depression Scale (Cox, Holden, & Sagovsky, 1987; assessing postpartum depression) and the General Anxiety Disorder – 7 (Spitzer, Kroenke, Williams, & Lowe, 2006; assessing generalized anxiety). This outcome will be measured during an in-home interview when the study child is approximately 6 months old.

8. **Mother parenting behaviors.** This outcome will be measured based on mother self-report of positive and negative parenting behaviors toward the study child (Durham Family Initiative, 2008; Lounds, J.J., Borkowski, J.G. & Whitman, T.L., 2004; Straus, M. A., Hamby, S. L., Finkelhor, D., & Runyan, D., 1995). This outcome will be measured during an in-home interview when the study child is approximately 6 months old.

9. **Mother infant intentionality beliefs.** This outcome will be measured based on mother self-report regarding the extent to which infants can intentionally engage in negative behaviors (Feldman & Reznick, 1996). This outcome will be measured during an in-home interview when the study child is approximately 6 months old.

10. **Father-child relationship quality.** This outcome will be measured based on mother report of father involvement in caring for the study child (Center for Research on Child Wellbeing, 2008). This outcome will be measured during an in-home interview when the study child is approximately 6 months old.

11. **Family connections to community services and resources.** This outcome will be measured based on mother self-reported family use (or not) of various formal and informal services and resources in the Durham, NC community. This outcome will be measured during an in-home interview when the study child is approximately 6 months old.

12. **Out of home childcare utilization.** This outcome will be measured based on mother self-reported use (or not) of out-of-home childcare for the study child. This outcome will be measured during an in-home interview when the study child is approximately 6 months old.

5. Populations and Subgroups to be Analyzed

5.1 Populations

1. **Intent-to-treat (ITT).** All randomized study subjects that provided written consent to participate in the outcome evaluation interview at infant age 6 months. To avoid potential contamination of the study design, any family that had previous participated in Durham Connects prior to the start of this RCT were removed from the sample. Additionally, to avoid violating assumptions of linear regression models, for participating mothers who gave birth to multiples (e.g., twins), one child was randomly chosen for inclusion in the study analyses.

5.2 Subgroups

One set of subgroup analyses will be conducted based on preliminary analyses that suggest baseline differences exist between the intervention and control groups based on the total number of medical risks at birth.

1. **Infant total medical risk at birth.** Subgroup analyses will examine differences in outcomes based on the total number of medical risks at birth, a 0-5 count variable summing the following: 1) born at less than 2500 grams; 2) less than 37 weeks gestational age, 3) birth complications, not specified; 4) substance exposure in utero; and 5) other risk noted, not specified.

6. Analyses

Descriptive statistic (mean, standard deviation, frequency, and percent) will be used to describe the birth risks, demographic and socioeconomic characteristics, and primary and secondary outcomes for the whole sample. Then, the birth risks and demographic and socioeconomic characteristics at baseline will be compared between children and their mothers in the treatment group and those in the control group by using chi-squared tests for categorical variables and using t-tests for continuous variables. If any imbalance at baseline exists between the two groups, a regression adjustment will be used to create adjusted means in outcomes for each group by controlling birth risks and demographic and socioeconomic characteristics. Stata 14.2 will be used for all statistical analysis.

6.1 Primary Outcome

A Poisson model will be applied when the outcomes are number of assessments and substantiations. The independent variable is treatment status (treatment=1). The covariates include birth risks, demographic, and socioeconomic characteristics. An adjusted mean of the outcomes in each group will be reported as well as a group difference and its confidence interval.

6.2 Secondary Outcomes

A linear model will be applied if the outcomes are continuous variables. A Poisson model will be applied if the outcomes are count variables. A logistic model will be applied if the outcomes are dichotomous variables. The covariates include birth risks, demographic, and socioeconomic characteristics. An adjusted mean of the outcomes in each group will be reported as well as a group difference and its confidence interval.

7. Missing Data

Missing data issue is commonly seen in social science research. Our missing data could come from item nonresponse and missingness in administrative data and survey. Multiple imputation techniques can reduce non-response bias, improve efficiency, and increase statistics power in parameter estimates, as compared to listwise deletion. Therefore, multiple imputation procedures will be applied to eliminate missing data in this study. The number of imputation will be 10.
