# Supplementary material for: Effect of a Community Agency–Administered Nurse Home Visitation Program on Program Use and Maternal and Infant Health Outcomes: A Randomized Clinical Trial
Source: JAMA Netw Open. 2019 Nov 1;2(11):e1914522. doi: 10.1001/jamanetworkopen.2019.14522 (PMC6826644; doi:10.1001/jamanetworkopen.2019.14522)
Supplement: Supplement 2. — Data Sharing Statement [file jamanetwopen-2-e1914522-s002.pdf]

Data Sharing Statement

Dodge KA, Goodman WB, Bai Y, O'Donnell K, Murphy RA. Effect of a community agency–administered nurse home visitation program on program use and maternal and infant health outcomes: a randomized clinical trial. *JAMA Netw Open*. 2019;2(11):e1914522. 10.1001/jamanetworkopen.2019.14522

Data
Data available: Yes
Data types: Other (please specify)
Additional Information: De-identified, upon receipt of an approved IRB and plan
How to access data: Data will be available by direct transfer.
When available: With publication

Supporting Documents
Document types: Other (please specify)
Additional Information: Codebooks
How to access documents: Codebooks
When available: With publication

Additional Information
Who can access the data: Researchers with an IRB approval and proposal.
Types of analyses: Any
Mechanisms of data availability: After approval
Any additional restrictions: None
